# Synthesis, crystal structure and Hirshfeld surface analysis of [Cu(H_2_*L*)_2_(μ-Cl)CuCl_3_]·H_2_O [H_2_*L* = 2-hy­droxy-*N*′-(propan-2-yl­idene)benzohydrazide]

**DOI:** 10.1107/S2056989024007941

**Published:** 2024-08-20

**Authors:** Imededdine Boulguemh, Asma Lehleh, Chahrazed Beghidja, Adel Beghidja

**Affiliations:** a Unité de Recherche de Chimie de l'Environnement et Moléculaire Structurale (CHEMS) Université Constantine 1 - Frères Mentouri 25017 Constantine Algeria; University of Missouri-Columbia, USA

**Keywords:** hydrazone, crystal structure, copper complexes, Hirshfeld surface, hydrogen bonds

## Abstract

The present study focuses on the synthesis and structural characterization of a novel dinuclear Cu^II^ complex, [tri­chlorido­copper(II)]-μ-chlorido-{bis­[2-hy­droxy-*N*′-(propan-2-yl­idene)benzohydrazide]copper(II)} monohydrate, [Cu_2_Cl_4_(C_10_H_12_N_2_O_2_)_2_]·H_2_O or [Cu(H_2_*L*)_2_(μ-Cl)CuCl_3_]·H_2_O [H_2_*L* = 2-hy­droxy-*N*′-(propan-2-yl­idene)benzohydrazide]. The complex crystallizes in the monoclinic space group *P*2_1_/*n* with one mol­ecule of water, which forms inter­actions with the ligands. The first copper ion is penta-coordinated to two benzohydrazine-derived ligands *via* two nitro­gen and two oxygen atoms, and one bridging chloride, which is also coordinated by the second copper ion alongside three terminal chlorines in a distorted tetra­hedral geometry. The arrangement around the first copper ion exhibits a distorted geometry inter­mediate between trigonal bipyramidal and square pyramidal. In the crystal, chains are formed *via* inter­molecular inter­actions along the *a-*axis direction, with subsequent layers constructed through hydrogen-bonding inter­actions parallel to the *ac* plane, and through slipped π–π stacking inter­actions parallel to the *ab* plane, resulting in a three-dimensional network. The inter­molecular inter­actions in the crystal structure were qu­anti­fied and analysed using Hirshfeld surface analysis. Residual electron density from disordered methanol mol­ecules in the void space could not be reasonably modelled, thus a solvent mask was applied.

## Abstract 2

The synthesis and structural characterization of a dinuclear Cu^II^ complex, [tri­chlorido­copper(II)]-μ-chlorido-{bis­[2-hy­droxy-*N*′-(propan-2-yl­idene)benzohydrazide]copper(II)} monohydrate, [Cu_2_Cl_4_(C_10_H_12_N_2_O_2_)_2_]·H_2_O or [Cu(H_2_L)_2_(μ-Cl)CuCl_3_]·H_2_O is reported

## 1. Chemical context

Schiff bases are organic compounds that have important applications in many areas of chemistry, including organic synthesis and inorganic chemistry (Sinicropi *et al.*, 2022 ▸). Over the years, Schiff bases have gained a lot of popularity as chelating ligands in coordination chemistry with transition metals, due to their versatility and ability to act as multiple linkers and their stability under various oxidizing and reducing conditions (DeepikaVerma *et al.*, 2023 ▸). These ligands make excellent coordination mol­ecules and can show variety in structures with metal complexes (Guo *et al.*, 2011 ▸), thus leading to a variety of properties (DeepikaVerma *et al.*, 2023 ▸).

Hydrazone ligands constitute a distinct category of Schiff bases, arising from the condensation reaction involving hydrazine and either an aldehyde or a ketone in the presence of an acid or a base. The literature has reported that coordin­ation complexes formed between hydrazones and metals can be used in several areas, such as in catalysis for various reactions (Dile *et al.*, 2016 ▸), as materials for gas adsorption (Roztocki *et al.*, 2016 ▸), for the detection of heavy metals in the environment (Sharma *et al.*, 2019 ▸), in electrochemistry (Toledano-Magaña *et al.*, 2015 ▸) and in mol­ecular magnetism (Sadhukhan *et al.*, 2018 ▸). In addition, these complexes are widely studied in pharmaceutical chemistry, (Haider & Khan, 2022 ▸) due to their potential as bioactive compounds, especially as anti­cancer (Šermukšnytė *et al.*, 2022 ▸; Gaur *et al.*, 2022 ▸), anti­tuberculosis (Mathew *et al.*, 2015 ▸; Teneva *et al.*, 2023 ▸) and anti­fungal agents (Kajal *et al.*, 2014 ▸) (Yankin *et al.*, 2022 ▸), as well as for the design of drugs against Alzheimer’s disease (Boulguemh *et al.*, 2020 ▸) and Parkinson’s disease (Kondeva-Burdina *et al.*, 2022 ▸).
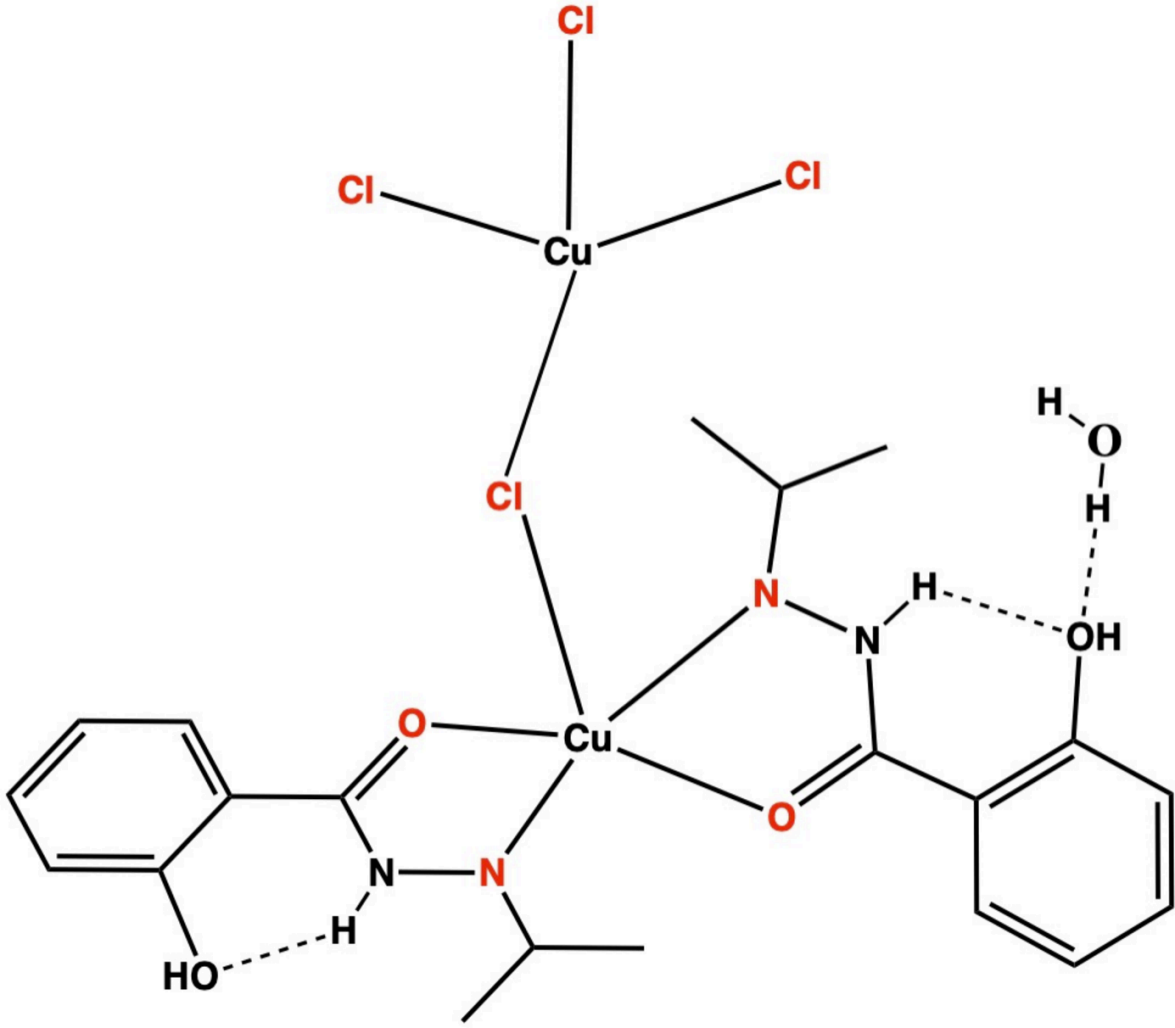


As a continuation of our research on the synthesis and the study of the biological and magnetic properties of new Schiff base-type ligands and their complexes (Ouilia *et al.*, 2012 ▸; Boussadia *et al.*, 2020 ▸; Boulguemh *et al.*, 2020 ▸), we report here the synthesis, structural characterization and Hirshfeld surface analysis of a new dinuclear copper(II) complex [Cu(H_2_*L*)_2_(μ-Cl)CuCl_3_]·H_2_O with a hydrazine ligand (H_2_*L* = 2-hy­droxy-*N*′-(propan-2-yl­idene)benzohydrazide).

## 2. Structural commentary

**Figure 1:**
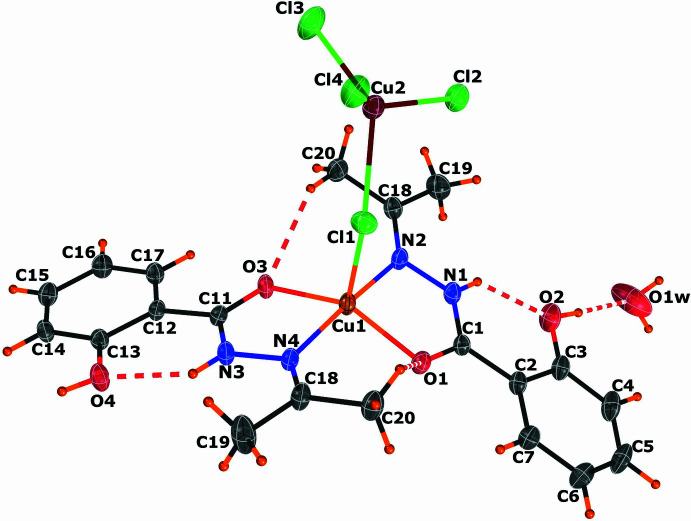
The title compound showing the atom-labelling scheme with ellipsoids drawn at the 50% probability level and H atoms shown as small spheres of arbitrary radii.

The asymmetric unit of the title compound, which comprises a dinuclear Cu^II^ complex and one water solvation mol­ecule, is illustrated in Fig. 1 ▸. The first copper ion Cu1 is in penta­coordinated environment with trigonality index parameter *τ_5_* = 0.516. The tau value for penta­coordinated complexes is calculated using the equation elaborated by Addison *et al.* (1984 ▸): *τ_5_* = (β − α)/60, where α and β are the largest basal angles. *τ_5_* equals 1 for an ideal trigonal bipyramid and 0 for a square-pyramidal coordination. The coordination geometry around the Cu1 ion lies between a distorted trigonal bipyramidal and square pyramidal. The copper ion Cu1 is coordin­ated to the two carbonyl oxygen atoms O1 and O3, and the two imine nitro­gens N2 and N4 from two bidentate chelating H_2_*L* ligands. The fifth coordination site is occupied by a bridging chloride Cl1 with a Cu1—Cl1 distance of 2.5001 (14) Å, consistent with literature values (Comba *et al.*, 1988 ▸). The Cu1—O bond lengths are Cu1—O1 = 1.971 (3) Å and Cu1—O3 = 1.959 (3) Å, while the Cu1—N2 and Cu1—N4 bond lengths are 1.999 (4) and 2.009 (4) Å, respectively. The distorted tetra­hedral site around the second copper ion, Cu2, is occupied by three terminal chloride ions, Cl2, Cl3, and Cl4 and a bridging chloride ion Cl1. The terminal Cu—Cl bond distances range from 2.2209 (16) to 2.2601 (12) Å, while the Cu—Cl bridging bond is slightly longer, with a Cu2—Cl1 distance of 2.2897 (15) Å (Table 1 ▸). These distances are comparable to those observed for other tetra­chloro­metallate (Vasilevesky *et al.*, 1991 ▸; Ramos Silva *et al.*, 2005 ▸; Comba *et al.*, 1988 ▸). The geometry index for tetra­coordinated copper ions, *τ_4_*, is calculated as [360° − (α + β)]/141° (Yang *et al.*, 2007 ▸), inspired by the *τ_5_* index for five-coordinate complexes developed by Addison and Reedijk (Addison *et al.*, 1984 ▸). The values of *τ_4_* range from 1 for a perfect tetra­hedral geometry to 0 for a perfect square-planar geometry. For the tetra­coordinated coordination geometry around the Cu2 ion, *τ_4_* = 0.61, indicating a very distorted tetra­hedral geometry (Yang *et al.*, 2007 ▸)*.* This distortion has been noted in numerous salts containing [CuCl_4_]^2−^ ions, with some displaying thermochromic properties attributed to the deformation of tetra­chloro­metallate ions in response to temperature changes (Willett *et al.*, 1974 ▸).

The Cu1—Cl1—Cu2 bridging angle of 135.00 (5)° is larger than those observed in the literature for yellow terminal tetra­chloro­metallate ligands (Ramos Silva *et al.*, 2005 ▸). However, the inter­metallic Cu⋯Cu distance observed in the title compound [4.426 (8) Å] is within the range observed for similar compounds (Comba *et al.*, 1988 ▸; Ramos Silva *et al.*, 2005 ▸). Some correlations between the magnetic and structural parameters for mono-μ-chloro–copper chains have been observed, while the magnetic and structural data suggest a limited number of exchange pathways (van Albada *et al.*, 2004 ▸; Alves *et al.*, 2009 ▸). However, following these correlations, overall ferromagnetic behaviour can be expected for values of the quotient φ/*R* (where φ is the Cu—Cl—Cu bridge angle and *R* is the Cu—Cl long bond length) lower than approximately 40 and higher than 57, whereas anti­ferromagnetic behaviour is observed when this quotient φ/*R* is between these two values. However, the terminal halometallate counter-ion has no impact on the nature of the inter­action. In the title compound, the Cu—Cl—Cu bond angle is 135.00 (5)°, with Cu—Cl distances of 2.5001 (14) and 2.2897 (15) Å, resulting in φ/*R* ratios of 54 and 59, respectively. These values suggest anti­ferromagnetic behaviour.

## 3. Supra­molecular features

In the crystal, the supra­molecular network consists of an extensive set of intra- and inter­molecular hydrogen-bonding interactions (numerical details are given in Table 2 ▸). Two intra­molecular hydrogen bonds are formed between the imine N1 and N3 atoms and phenolic O2, O4 atoms of the ligand *via* the respective hydrogen atoms H1 and H3 (Fig. 1 ▸). While the carbon donor atoms C10 and C20 of the methyl groups are involved in hydrogen bonds with the acceptor atoms O3 and O1, respectively, of the carbonyl groups *via* the H10*C* and H20*C* atoms (Fig. 1 ▸). The solvent water mol­ecule is linked to the complex mol­ecule *via* the oxygen atom O2 of the phenolic group by a O2—H2⋯O1*W* hydrogen bond (Fig. 1 ▸).

**Figure 2:**
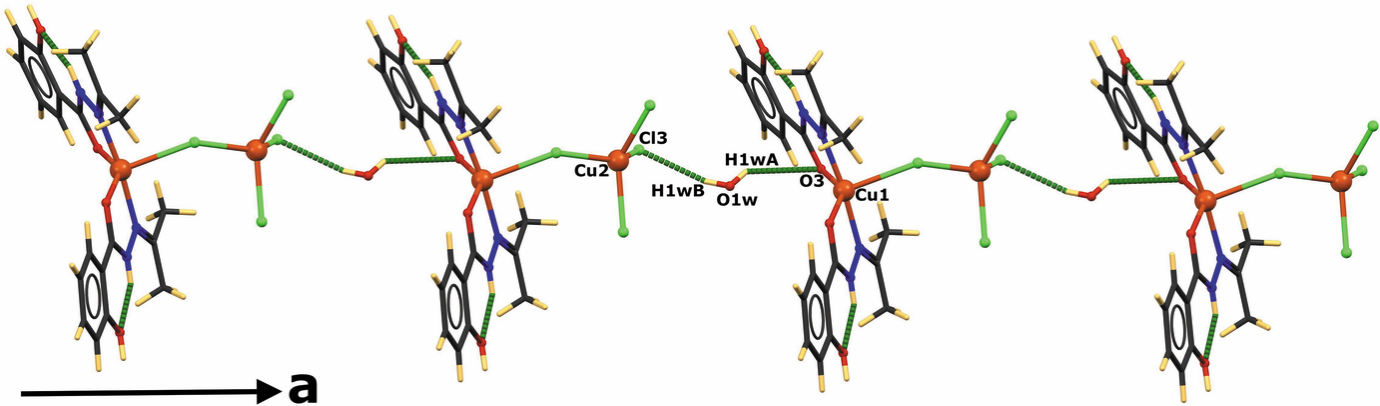
Partial view of the crystal structure showing the inter­molecular hydrogen bonds (indicated by green dashed lines) forming infinite chains propagating along the *a-*axis direction.

**Figure 3:**
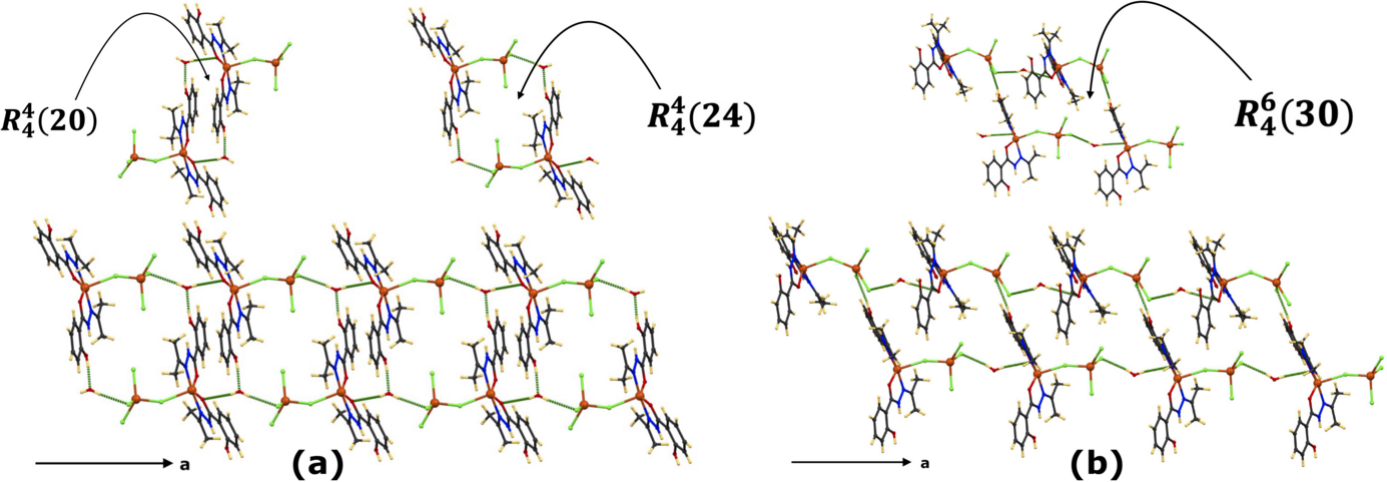
Mol­ecular view of the arrangement of chains (*a*) *via R*^4^_4_(20) and 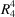
(24) rings and (*b*) 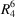
(30) rings along the *ac* plane. Hydrogen bonds are shown as dashed green lines.

The complex mol­ecules are connected *via* two hydrogen bonds involving the water mol­ecule, O1*W*—H1*WB*⋯Cl3 and O1*W*—H1*WA*⋯O3, leading to chains propagating along the *a*-axis direction (Fig. 2 ▸). The two-dimensional arrangement parallel to the *ac* plane is established by connecting two adjacent chains through two types of patterns. The first arrangement is formed by a succession of 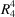
(20) and 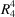
(24) rings, and the second one through a succession of 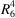
(30) rings (Fig. 3 ▸). The first two ring structures are formed by two water and two complex mol­ecules, except for the third ring, which is formed by two solvent water and four complex mol­ecules.

In the first arrangement, the two water mol­ecules act as acceptor and donor, forming 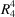
(20) and 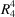
(24) rings. O2—H2⋯O1*W*—H1*WA*⋯O3 inter­actions are observed in the first ring and O2—H2⋯O1*W*—H1*WB*⋯Cl3 in the second ring (Fig. 3 ▸*a*). The second arrangement of inter­connected chains is generated by a succession of 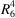
(30) rings, where the two water mol­ecules act as donors in Cl3⋯ H1*WB* —O1*W*—H1*WA*⋯O3 inter­actions, and two phenol donor groups in O4—H4⋯Cl2 inter­actions (Fig. 3 ▸*b*).

**Figure 4:**
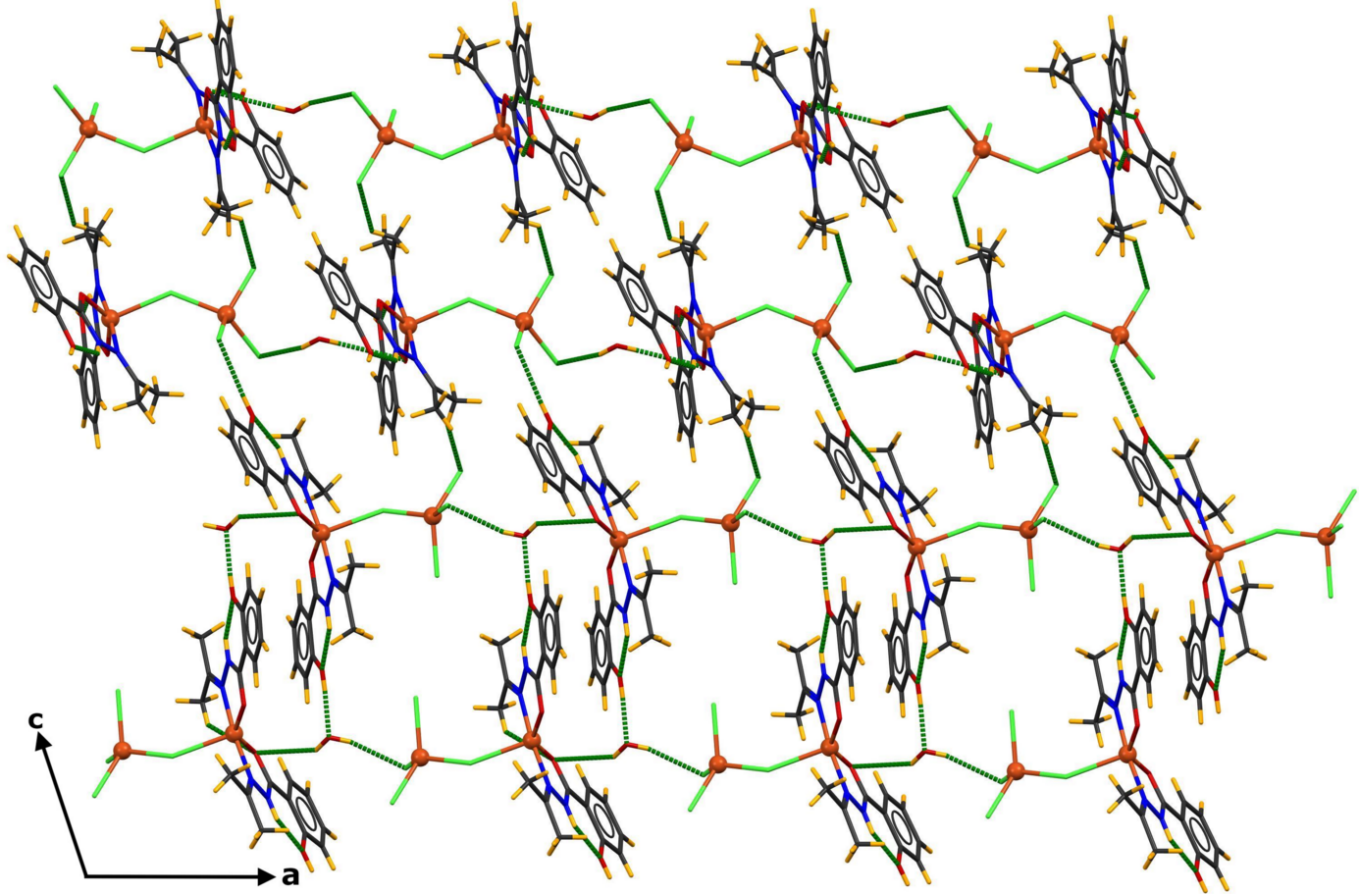
Crystal packing of the title compound shown in a projection of the two-dimensional network connected through hydrogen bonds (shown as dashed green lines).

**Figure 5:**
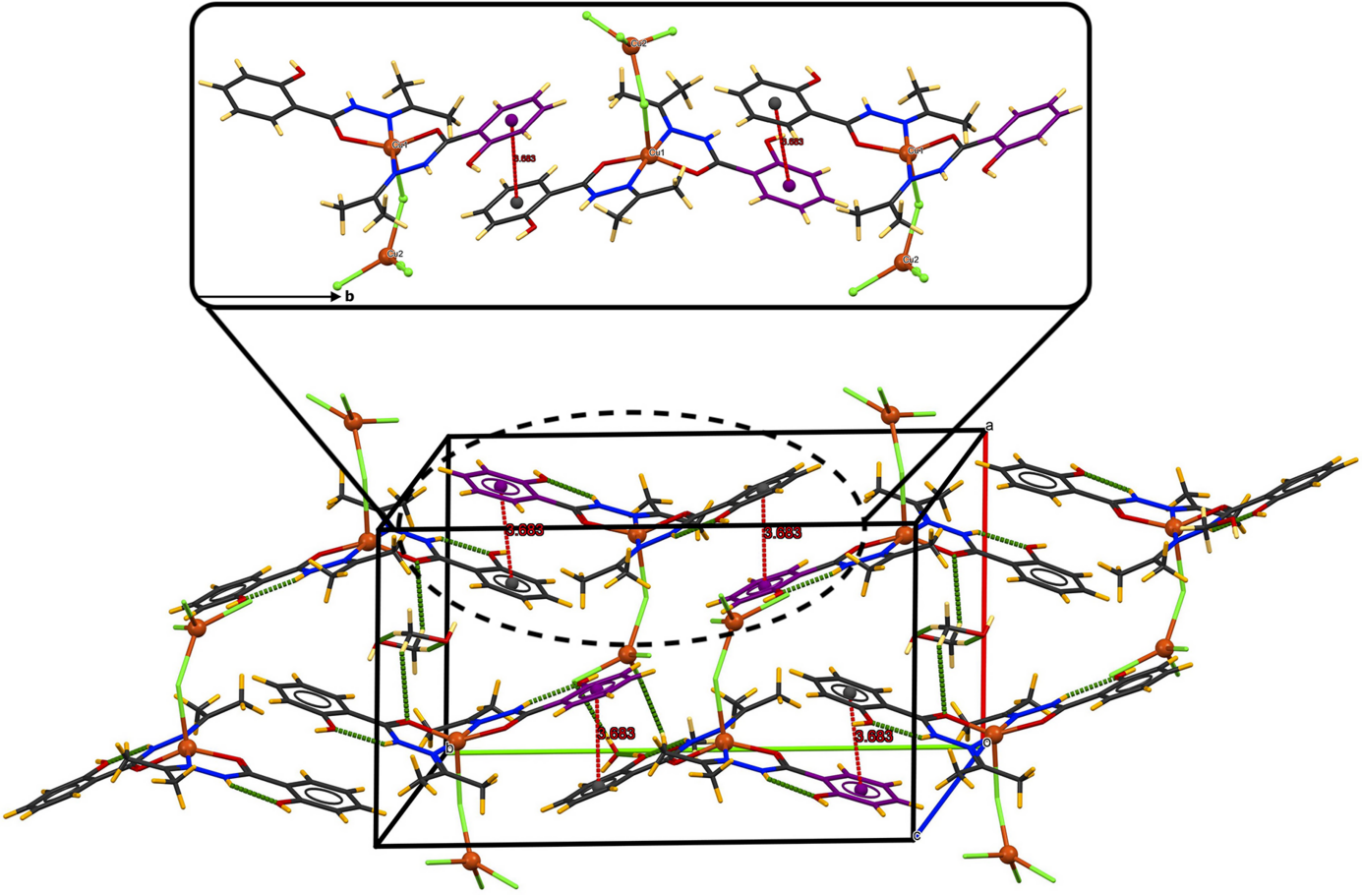
Crystal packing of the title compound showing the 3D network and the chains parallel to the *b* axis formed by π–π stacking inter­actions between the aromatic rings of the ligands (shown in red).

The junction between the resulting two double chains *via* hydrogen bonds O4—H4⋯Cl2 and C10—H10*A*⋯Cl4 establishes two-dimensional layers parallel to the *ac* plane (Fig. 4 ▸). Slipped π–π stacking inter­actions are also observed in this structure, involving the aromatic rings of the ligands with an inter­centroid distance *Cg*1⋯·*Cg*2(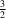 − *x*, −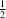 + *y*, 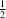 − *z*) of 3.683 (3) Å (where *Cg*1 and *Cg*2 are the centroids of the C2–C7 and C12–C17 rings, respectively), resulting in a three-dimensional network by linking chains along the *b* axis (Fig. 5 ▸).

## 4. Database survey

A search of the Cambridge Structural Database (CSD version 5.45, updated in November 2023; Groom *et al.*, 2016 ▸), revealed that crystal structures have been reported for complexes of several hydrazone derivatives with various metal ions, such as copper (Balsa *et al.*, 2021 ▸), zinc (Dasgupta *et al.*, 2020 ▸), cadmium (Govindaiah *et al.*, 2021 ▸), cobalt (Han *et al.*, 2020 ▸), magnesium (Khandar *et al.*, 2019 ▸). Only one complex based on copper and benzoic acid, 2-(1-methyl­ethyl­idene)hydrazide has been reported (Mohamad *et al.*, 2019 ▸). No complexes containing two copper ions connected to each other by a chlorine atom and coordinated to two mol­ecules of acetone hydrazone have been documented in the CSD.

To the best of our knowledge, there are only a few examples of asymmetric binuclear copper-based complexes reported in the CSD with some instances where a copper complex is bridged by any type of tetra­metallate (Barz *et al.*, 1998 ▸; Shi *et al.*, 2014 ▸; Alves *et al.*, 2014 ▸; Kaur *et al.*, 2019 ▸; Singh *et al.*, 2014 ▸; Comba *et al.*, 1988 ▸; Ramos Silva *et al.*, 2005 ▸).

## 5. Hirshfeld surface analysis

**Figure 6:**
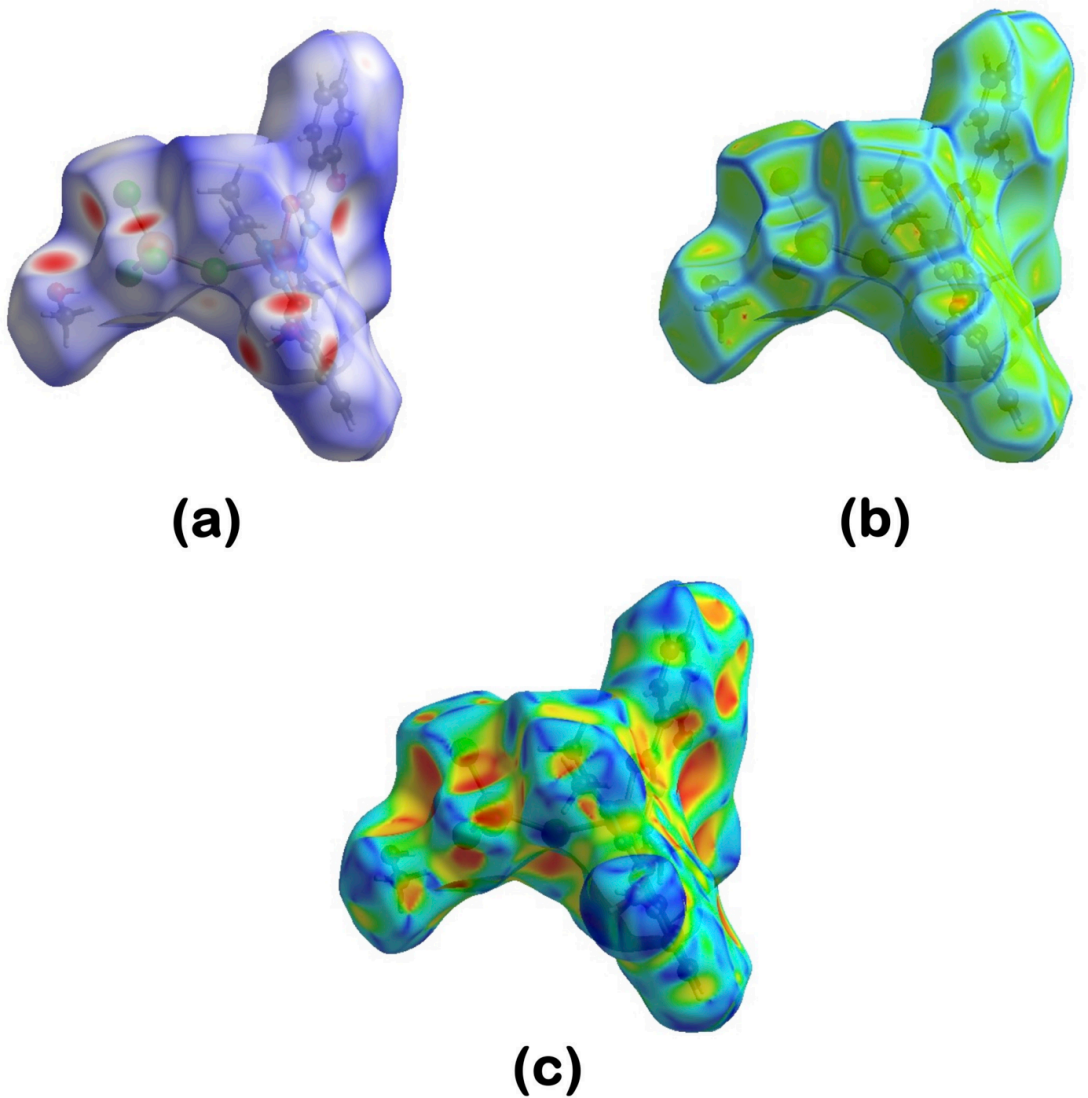
A view of the Hirshfeld surface mapped over (*a*) *d*_norm_, (*b*) shape-index and (*c*) curvedness.

**Figure 7:**
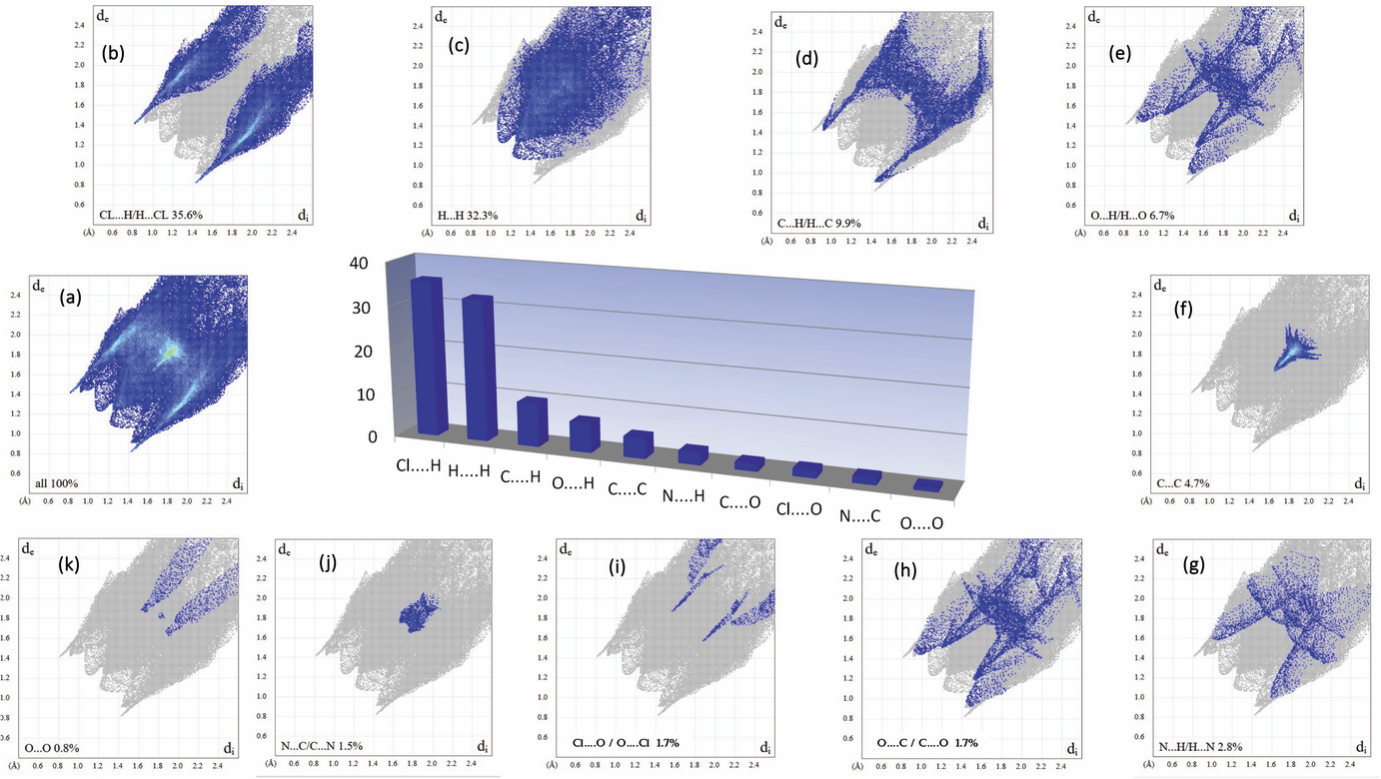
Two-dimensional fingerprint plots for the title compound, showing (*a*) all inter­actions, and delineated into (*b*) Cl⋯H/H⋯Cl, (*c*) H⋯H, (*d*) C⋯H/ H⋯C, (*e*) O⋯H/ H⋯O, (*f*) C⋯C, (*g*) N⋯H/H⋯N, (*h*) O⋯C/C⋯O, (*i*) N⋯C/C⋯N and (*j*) O⋯O inter­actions. The *d*_i_ and *d*_e_ values are the closest inter­nal and external distances (in Å) from given points on the Hirshfeld surface.

For further characterization of the inter­molecular inter­actions in the title compound, we carried out a Hirshfeld surface (HS) analysis (Spackman & Jayatilaka, 2009 ▸) using *Crystal Explorer 21* (Spackman *et al.*, 2021 ▸) and generated the associated two-dimensional fingerprint plots (McKinnon *et al.*, 2007 ▸). The HS of the title compound mapped over *d*_norm_ in the range 0.4396 to +2.3676 a.u. is illustrated in Fig. 6 ▸ using colour to indicate contacts that are shorter (red areas), equal to (white areas), or longer than (blue areas) the sum of the van der Waals radii (Ashfaq *et al.*, 2021 ▸). The red spots on the surface mapped over *d*_norm_ (Fig. 6 ▸*a*) indicate the involvement of atoms in hydrogen-bonding inter­actions. The HS mapped over shape-index (Fig. 6 ▸*b*) is used to check for the presence of inter­actions such as C—H⋯π and π–π stacking (Ashfaq *et al.*, 2021 ▸). The existence of adjacent red and blue triangular regions around the aromatic rings conforms to the presence of π–π stacking inter­actions in the title compound (Fig. 6 ▸*b*), and the curvedness plots (Fig. 6 ▸*c*) show flat surface patches characteristic of planar stacking. The two-dimensional fingerprint plots provide unique information about the non-covalent inter­actions and the crystal packing in terms of the percentage contribution of the inter­atomic contacts (Spackman & McKinnon, 2002 ▸; Ashfaq *et al.*, 2021 ▸). Fig. 7 ▸ shows the two-dimensional fingerprint plot for the overall inter­actions with their relative contributions to the Hirshfeld surface. The most important inter­atomic contact is H⋯Cl as it makes the highest contribution to the crystal packing (35.6%, Fig. 7 ▸*b*). The other major contributor is H⋯H inter­actions (32.3%, Fig. 7 ▸*c*). Other inter­actions contributing less to the crystal packing are C⋯H (9.9%, Fig. 7 ▸*d*), O⋯H (6.7%, Fig. 7 ▸*e*), C⋯C (4.7%, Fig. 7 ▸*f*), N⋯H (2.8%, Fig. 7 ▸*g*), C⋯O (1.7%, Fig. 7 ▸*h*), Cl⋯O (1.7%, Fig. 7 ▸*i*), N⋯C (1.5%, Fig. 7 ▸*j*) and O⋯O (0.8%, Fig. 7 ▸*k*). Other contacts make a contribution of 2.3% in total and are not discussed in this work.

## 6. Synthesis and crystallization

A mixture of CuCl_2_·2H_2_O (0.170 g, 1mmol) with salicylhydrazide (0.304 g, 2 mmol) and NaOH (0.08 g, 2 mmol), was dissolved in 10 mL of a mixed methanol/acetone (3/1) solution then stirred for 2 h at room temperature. Yellow crystals suitable for X-ray analysis were obtained after 5 days in (0.022 g, 52%).

## 7. Refinement

Crystal data, data collection and structure refinement details are summarized in Table 3 ▸. H atoms were placed in calculated positions with C—H = 0.93–0.95 Å, N—H = 0.86 Å, O—H = 0.82–0.85 Å and refined using a riding model with *U*_iso_(H) = 1.2–1.5*U*_eq_(C,N,O). A solvent mask was calculated *via* the SQUEEZE routine in *PLATON* (Spek, 2015 ▸, 2020 ▸) and 120 electrons were found in a volume of 234 Å^3^ in two voids per unit cell. This is consistent with the presence of 1[H_2_O], 1.5[CH_3_OH] per formula unit, which account for 122 electrons per unit cell. 

## Supplementary Material

Crystal structure: contains datablock(s) global, I. DOI: 10.1107/S2056989024007941/ev2007sup1.cif

Structure factors: contains datablock(s) I. DOI: 10.1107/S2056989024007941/ev2007Isup2.hkl

CCDC reference: 2377149

Additional supporting information:  crystallographic information; 3D view; checkCIF report

## Figures and Tables

**Table 1:** Selected geometric parameters (Å, °)

Cu1—Cl1	2.5001 (14)	Cu2—Cl1	2.2897 (15)
Cu1—O1	1.971 (3)	Cu2—Cl2	2.2601 (12)
Cu1—O3	1.959 (3)	Cu2—Cl3	2.2209 (16)
Cu1—N2	1.999 (4)	Cu2—Cl4	2.2269 (13)
Cu1—N4	2.009 (4)		
			
Cl1—Cu1—O1	108.38 (10)	N2—Cu1—N4	173.85 (15)
Cl1—Cu1—O3	108.44 (11)	Cl1—Cu2—Cl2	99.52 (5)
Cl1—Cu1—N2	96.87 (11)	Cl1—Cu2—Cl3	135.68 (6)
Cl1—Cu1—N4	89.26 (11)	Cl1—Cu2—Cl4	94.92 (5)
O1—Cu1—O3	143.06 (14)	Cl2—Cu2—Cl3	98.34 (5)
O1—Cu1—N2	81.01 (13)	Cl2—Cu2—Cl4	137.89 (5)
O1—Cu1—N4	96.61 (13)	Cl3—Cu2—Cl4	98.32 (5)
O3—Cu1—N2	97.49 (13)	Cu1—Cl1—Cu2	135.00 (5)
O3—Cu1—N4	80.97 (12)		

**Table 2:** Hydrogen-bond geometry (Å, °)

*D*—H⋯*A*	*D*—H	H⋯*A*	*D*⋯*A*	*D*—H⋯*A*
O2—H2⋯O1*W*	0.82	1.79	2.606 (6)	171
N1—H1⋯O2	0.86	1.94	2.597 (5)	132
N3—H3⋯O4	0.86	1.96	2.612 (5)	132
O1*W*—H1*WA*⋯O3^i^	0.85	2.53	3.375 (6)	170
O1*W*—H1*WA*⋯N3^i^	0.85	2.59	3.303 (7)	142
O1*W*—H1*WB*⋯Cl3^ii^	0.85	2.38	3.195 (6)	160
O4—H4⋯Cl2^iii^	0.82	2.40	3.215 (3)	175
C7—H7⋯O1	0.93	2.44	2.762 (5)	1
C10—H10*C*⋯O3	0.96	2.35	3.099 (6)	135
C20—H20*C*⋯O1	0.96	2.41	3.043 (5)	123

Symmetry codes: (i) 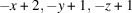; (ii) 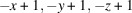; (iii) 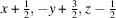.

**Table 3:** Experimental details

Crystal data	
Chemical formula	[Cu_2_Cl_4_(C_10_H_12_N_2_O_2_)_2_]·2H_2_O·1.5CH_4_O
*M*_r_	737.40
Crystal system, space group	Monoclinic, *P*2_1_/*n*
Temperature (K)	273
*a*, *b*, *c* (Å)	11.6514 (4), 20.1507 (8), 12.8149 (4)
β (°)	110.858 (2)
*V* (Å^3^)	2811.56 (18)
*Z*	4
Radiation type	Mo *K*α
μ (mm^−1^)	1.94
Crystal size (mm)	0.14 × 0.12 × 0.09

Data collection	
Diffractometer	Bruker APEXII CCD
Absorption correction	Multi-scan (*SADABS*; Krause *et al.*, 2015 ▸)
*T*_min_, *T*_max_	0.673, 0.745
No. of measured, independent and observed [*I* > 2σ(*I*)] reflections	21699, 5708, 3843
*R*_int_	0.055
(sin θ/λ)_max_ (Å^−1^)	0.626

Refinement	
*R*[*F*^2^ > 2σ(*F*^2^)], *wR*(*F*^2^), *S*	0.049, 0.146, 1.06
No. of reflections	5708
No. of parameters	324
H-atom treatment	H-atom parameters constrained
Δρ_max_, Δρ_min_ (e Å^−3^)	0.71, −0.53

Computer programs: *APEX2* and *SAINT* (Bruker, 2013 ▸), *SHELXT2018/3* (Sheldrick, 2015*a* ▸), *SHELXL2018/3* (Sheldrick, 2015*b* ▸), *Mercury* (Macrae *et al.*, 2020 ▸) and *PLATON* (Spek, 2020 ▸).

## supplementary crystallographic information

### [Trichloridocopper(II)]-µ-chlorido-{bis[2-hydroxy-N'-(propan-2-ylidene)benzohydrazide]copper(II)} monohydrate Crystal data

**Table:**

[Cu_2_Cl_4_(C_10_H_12_N_2_O_2_)_2_]·2H_2_O·1.5CH_4_O	*F*(000) = 1360
*M**_r_* = 737.40	*D*_x_ = 1.704 Mg m^−^^3^
Monoclinic, *P*2_1_/*n*	Mo *K*α radiation, λ = 0.71073 Å
Hall symbol: -P 2yn	Cell parameters from 3635 reflections
*a* = 11.6514 (4) Å	θ = 2.6–23.4°
*b* = 20.1507 (8) Å	µ = 1.94 mm^−^^1^
*c* = 12.8149 (4) Å	*T* = 273 K
β = 110.858 (2)°	Block, yellow
*V* = 2811.56 (18) Å^3^	0.14 × 0.12 × 0.09 mm
*Z* = 4	

### [Trichloridocopper(II)]-µ-chlorido-{bis[2-hydroxy-N'-(propan-2-ylidene)benzohydrazide]copper(II)} monohydrate Data collection

**Table:**

Bruker APEXII CCD diffractometer	5708 independent reflections
Radiation source: MoKα	3843 reflections with *I* > 2σ(*I*)
Graphite monochromator	*R*_int_ = 0.055
Detector resolution: 18.4 pixels mm^-1^	θ_max_ = 26.4°, θ_min_ = 2.0°
φ and ω scans	*h* = −14→14
Absorption correction: multi-scan (SADABS; Krause *et al.*, 2015)	*k* = −21→25
*T*_min_ = 0.673, *T*_max_ = 0.745	*l* = −16→14
21699 measured reflections	

### [Trichloridocopper(II)]-µ-chlorido-{bis[2-hydroxy-N'-(propan-2-ylidene)benzohydrazide]copper(II)} monohydrate Refinement

**Table:**

Refinement on *F*^2^	0 restraints
Least-squares matrix: full	Hydrogen site location: mixed
*R*[*F*^2^ > 2σ(*F*^2^)] = 0.049	H-atom parameters constrained
*wR*(*F*^2^) = 0.146	W = 1/[Σ^2^(*F*O^2^) + (0.0793*P*)^2^] WHERE *P* = (*F*O^2^ + 2*F*C^2^)/3
*S* = 1.06	(Δ/σ)_max_ = 0.001
5708 reflections	Δρ_max_ = 0.71 e Å^−^^3^
324 parameters	Δρ_min_ = −0.53 e Å^−^^3^

### [Trichloridocopper(II)]-µ-chlorido-{bis[2-hydroxy-N'-(propan-2-ylidene)benzohydrazide]copper(II)} monohydrate Special details

**Table:**

Geometry. Bond distances, angles etc. have been calculated using the rounded fractional coordinates. All su's are estimated from the variances of the (full) variance-covariance matrix. The cell esds are taken into account in the estimation of distances, angles and torsion angles
Refinement. 1. Fixed Uiso At 1.2 times of: All C(H) groups, All N(H) groups At 1.5 times of: All C(H,H,H) groups, All O(H) groups, All O(H,H) groups2. Uiso/Uaniso restraints and constraints Uanis(C21) ~ Ueq: with sigma of 0.001 and sigma for terminal atoms of 0.002 Uanis(O5) ~ Ueq: with sigma of 0.001 and sigma for terminal atoms of 0.0023.a Free rotating group: O1W(H1WA,H1WB)3.b Aromatic/amide H refined with riding coordinates: N1(H1), N3(H3), C4(H4A), C5(H5), C6(H6), C7(H7), C14(H14), C15(H15), C16(H16), C17(H17)3.c Idealised Me refined as rotating group: C9(H9A,H9B,H9C), C10(H10A,H10B,H10C), C19(H19A,H19B,H19C), C20(H20A,H20B, H20C), C21(H21A,H21B,H21C)3.d Idealised tetrahedral OH refined as rotating group: O2(H2), O4(H4), O5(H5A).

### [Trichloridocopper(II)]-µ-chlorido-{bis[2-hydroxy-N'-(propan-2-ylidene)benzohydrazide]copper(II)} monohydrate Fractional atomic coordinates and isotropic or equivalent isotropic displacement parameters (Å^2^)

**Table:**

	*x*	*y*	*z*	*U*_iso_*/*U*_eq_	
Cu1	0.75452 (5)	0.61586 (3)	0.24378 (4)	0.0340 (2)	
Cu2	0.36066 (5)	0.63824 (3)	0.19419 (4)	0.0381 (2)	
Cl1	0.52667 (11)	0.61036 (6)	0.14739 (9)	0.0440 (4)	
Cl2	0.40338 (12)	0.57786 (6)	0.35215 (9)	0.0498 (4)	
Cl3	0.29411 (16)	0.72976 (7)	0.25093 (11)	0.0676 (5)	
Cl4	0.22690 (12)	0.63604 (6)	0.01947 (10)	0.0521 (4)	
O1	0.8210 (3)	0.52516 (15)	0.2546 (2)	0.0384 (10)	
O2	0.8882 (3)	0.42800 (17)	0.5540 (3)	0.0529 (11)	
O3	0.8022 (3)	0.70917 (14)	0.2734 (2)	0.0365 (10)	
O4	0.8657 (3)	0.81403 (15)	0.0217 (2)	0.0467 (10)	
N1	0.8076 (3)	0.52971 (17)	0.4240 (3)	0.0343 (11)	
N2	0.7577 (3)	0.59260 (17)	0.3964 (3)	0.0329 (11)	
N3	0.8051 (3)	0.70518 (17)	0.1001 (3)	0.0340 (11)	
N4	0.7711 (3)	0.63902 (16)	0.0973 (3)	0.0307 (11)	
C1	0.8365 (4)	0.4970 (2)	0.3474 (3)	0.0304 (12)	
O1W	0.9308 (4)	0.3537 (3)	0.7302 (4)	0.119 (2)	
C2	0.8862 (4)	0.4296 (2)	0.3687 (3)	0.0343 (12)	
C3	0.9105 (4)	0.3962 (2)	0.4704 (4)	0.0395 (14)	
C4	0.9573 (4)	0.3314 (2)	0.4824 (4)	0.0517 (17)	
C5	0.9782 (5)	0.3009 (3)	0.3947 (5)	0.0585 (19)	
C6	0.9532 (5)	0.3324 (3)	0.2941 (4)	0.0539 (17)	
C7	0.9075 (4)	0.3963 (2)	0.2808 (4)	0.0429 (17)	
C8	0.7218 (4)	0.6229 (2)	0.4683 (4)	0.0376 (14)	
C9	0.7308 (5)	0.5926 (3)	0.5773 (4)	0.0523 (17)	
C10	0.6685 (5)	0.6909 (2)	0.4417 (4)	0.0503 (17)	
C11	0.8230 (4)	0.7382 (2)	0.1949 (3)	0.0310 (12)	
C12	0.8656 (4)	0.8069 (2)	0.2065 (3)	0.0311 (12)	
C13	0.8874 (4)	0.8439 (2)	0.1218 (3)	0.0328 (12)	
C14	0.9286 (4)	0.9088 (2)	0.1416 (3)	0.0366 (14)	
C15	0.9450 (4)	0.9386 (2)	0.2436 (4)	0.0404 (14)	
C16	0.9226 (4)	0.9033 (2)	0.3277 (4)	0.0412 (16)	
C17	0.8840 (4)	0.8387 (2)	0.3086 (3)	0.0356 (14)	
C18	0.7490 (4)	0.6063 (2)	0.0057 (4)	0.0396 (14)	
C19	0.7628 (7)	0.6374 (3)	−0.0940 (4)	0.074 (3)	
C20	0.7147 (5)	0.5358 (2)	0.0012 (4)	0.0455 (16)	
H1	0.81939	0.51265	0.48846	0.0410*	
H2	0.89569	0.40190	0.60509	0.0800*	
H3	0.81424	0.72404	0.04332	0.0410*	
H4	0.87029	0.84159	−0.02370	0.0700*	
H4A	0.97419	0.30903	0.54971	0.0620*	
H5	1.00988	0.25806	0.40383	0.0700*	
H6	0.96696	0.31086	0.23543	0.0640*	
H7	0.89049	0.41769	0.21267	0.0520*	
H9A	0.66940	0.55874	0.56479	0.0790*	
H9B	0.71815	0.62622	0.62517	0.0790*	
H9C	0.81082	0.57334	0.61213	0.0790*	
H10A	0.72080	0.72206	0.49371	0.0750*	
H10B	0.58840	0.69159	0.44692	0.0750*	
H10C	0.66221	0.70267	0.36723	0.0750*	
H14	0.94531	0.93246	0.08641	0.0440*	
H15	0.97101	0.98253	0.25575	0.0490*	
H16	0.93375	0.92321	0.39606	0.0500*	
H17	0.86953	0.81523	0.36510	0.0430*	
H19A	0.70856	0.67474	−0.11693	0.1110*	
H19B	0.74279	0.60553	−0.15355	0.1110*	
H19C	0.84614	0.65190	−0.07606	0.1110*	
H20A	0.78488	0.50873	0.00817	0.0680*	
H20B	0.65036	0.52658	−0.06878	0.0680*	
H20C	0.68640	0.52596	0.06132	0.0680*	
H1WA	0.99407	0.33290	0.72978	0.1790*	
H1WB	0.88009	0.32335	0.73023	0.1790*	

### [Trichloridocopper(II)]-µ-chlorido-{bis[2-hydroxy-N'-(propan-2-ylidene)benzohydrazide]copper(II)} monohydrate Atomic displacement parameters (Å^2^)

**Table:**

	*U*^11^	*U*^22^	*U*^33^	*U*^12^	*U*^13^	*U*^23^
Cu1	0.0510 (4)	0.0231 (3)	0.0307 (3)	0.0007 (2)	0.0181 (2)	0.0037 (2)
Cu2	0.0512 (4)	0.0320 (3)	0.0345 (3)	0.0030 (2)	0.0193 (3)	−0.0012 (2)
Cl1	0.0426 (6)	0.0525 (7)	0.0402 (6)	0.0021 (5)	0.0188 (5)	0.0021 (5)
Cl2	0.0742 (9)	0.0429 (7)	0.0369 (6)	0.0065 (6)	0.0256 (6)	0.0047 (5)
Cl3	0.1140 (12)	0.0390 (7)	0.0589 (8)	0.0198 (7)	0.0419 (8)	−0.0043 (6)
Cl4	0.0633 (8)	0.0474 (8)	0.0386 (6)	0.0069 (6)	0.0094 (6)	−0.0016 (5)
O1	0.0520 (19)	0.0294 (16)	0.0341 (16)	0.0060 (14)	0.0156 (14)	0.0043 (13)
O2	0.073 (2)	0.044 (2)	0.0352 (18)	0.0046 (18)	0.0113 (17)	0.0127 (15)
O3	0.058 (2)	0.0241 (16)	0.0315 (15)	−0.0039 (14)	0.0209 (14)	0.0020 (12)
O4	0.083 (2)	0.0310 (17)	0.0346 (16)	−0.0098 (17)	0.0313 (17)	−0.0014 (13)
N1	0.045 (2)	0.0292 (19)	0.0293 (17)	0.0012 (16)	0.0140 (16)	0.0057 (15)
N2	0.043 (2)	0.0222 (18)	0.0344 (19)	−0.0030 (15)	0.0149 (16)	−0.0018 (14)
N3	0.050 (2)	0.0257 (19)	0.0301 (18)	−0.0050 (16)	0.0189 (16)	0.0010 (14)
N4	0.042 (2)	0.0212 (18)	0.0294 (18)	0.0000 (15)	0.0132 (15)	0.0016 (13)
C1	0.035 (2)	0.024 (2)	0.032 (2)	−0.0045 (17)	0.0116 (18)	−0.0001 (17)
O1W	0.069 (3)	0.195 (6)	0.096 (3)	0.026 (3)	0.032 (3)	0.098 (4)
C2	0.034 (2)	0.026 (2)	0.036 (2)	−0.0033 (18)	0.0040 (18)	0.0020 (17)
C3	0.043 (3)	0.030 (2)	0.037 (2)	−0.0046 (19)	0.004 (2)	0.0007 (18)
C4	0.047 (3)	0.032 (3)	0.059 (3)	0.002 (2)	−0.002 (2)	0.015 (2)
C5	0.051 (3)	0.028 (3)	0.080 (4)	0.007 (2)	0.003 (3)	−0.002 (3)
C6	0.055 (3)	0.040 (3)	0.062 (3)	0.008 (2)	0.015 (3)	−0.008 (2)
C7	0.049 (3)	0.035 (3)	0.042 (3)	0.002 (2)	0.013 (2)	−0.004 (2)
C8	0.040 (3)	0.031 (2)	0.040 (2)	−0.0055 (19)	0.012 (2)	−0.0015 (19)
C9	0.068 (3)	0.060 (3)	0.035 (3)	−0.002 (3)	0.026 (2)	−0.001 (2)
C10	0.070 (3)	0.034 (3)	0.057 (3)	−0.001 (2)	0.035 (3)	−0.007 (2)
C11	0.036 (2)	0.025 (2)	0.031 (2)	0.0014 (17)	0.0106 (18)	0.0023 (16)
C12	0.037 (2)	0.023 (2)	0.034 (2)	−0.0007 (17)	0.0137 (18)	0.0025 (16)
C13	0.042 (2)	0.028 (2)	0.029 (2)	−0.0001 (18)	0.0135 (18)	0.0033 (17)
C14	0.046 (3)	0.031 (2)	0.035 (2)	−0.006 (2)	0.017 (2)	0.0061 (18)
C15	0.048 (3)	0.028 (2)	0.042 (2)	−0.003 (2)	0.012 (2)	−0.0041 (19)
C16	0.054 (3)	0.035 (3)	0.034 (2)	−0.005 (2)	0.015 (2)	−0.0057 (19)
C17	0.046 (3)	0.032 (2)	0.030 (2)	−0.0027 (19)	0.0151 (19)	0.0025 (17)
C18	0.058 (3)	0.027 (2)	0.032 (2)	−0.001 (2)	0.014 (2)	0.0025 (18)
C19	0.138 (6)	0.052 (4)	0.037 (3)	−0.025 (4)	0.036 (3)	−0.003 (2)
C20	0.066 (3)	0.032 (3)	0.036 (2)	−0.005 (2)	0.015 (2)	−0.0033 (19)

### [Trichloridocopper(II)]-µ-chlorido-{bis[2-hydroxy-N'-(propan-2-ylidene)benzohydrazide]copper(II)} monohydrate Geometric parameters (Å, º)

**Table:**

Cu1—Cl1	2.5001 (14)	C11—C12	1.460 (6)
Cu1—O1	1.971 (3)	C12—C13	1.412 (6)
Cu1—O3	1.959 (3)	C12—C17	1.403 (5)
Cu1—N2	1.999 (4)	C13—C14	1.385 (6)
Cu1—N4	2.009 (4)	C14—C15	1.389 (6)
Cu2—Cl1	2.2897 (15)	C15—C16	1.391 (7)
Cu2—Cl2	2.2601 (12)	C16—C17	1.371 (6)
Cu2—Cl3	2.2209 (16)	C18—C19	1.482 (7)
Cu2—Cl4	2.2269 (13)	C18—C20	1.471 (6)
O1—C1	1.271 (5)	O1W—H1WB	0.8500
O2—C3	1.351 (6)	O1W—H1WA	0.8500
O3—C11	1.260 (5)	C4—H4A	0.9300
O4—C13	1.357 (5)	C5—H5	0.9300
N1—N2	1.387 (5)	C6—H6	0.9300
N1—C1	1.322 (5)	C7—H7	0.9300
N2—C8	1.293 (6)	C9—H9A	0.9600
O2—H2	0.8200	C9—H9C	0.9600
N3—C11	1.335 (5)	C9—H9B	0.9600
N3—N4	1.388 (5)	C10—H10B	0.9600
N4—C18	1.290 (6)	C10—H10C	0.9600
O4—H4	0.8200	C10—H10A	0.9600
N1—H1	0.8600	C14—H14	0.9300
C1—C2	1.464 (6)	C15—H15	0.9300
C2—C3	1.403 (6)	C16—H16	0.9300
C2—C7	1.407 (6)	C17—H17	0.9300
N3—H3	0.8600	C19—H19B	0.9600
C3—C4	1.402 (6)	C19—H19C	0.9600
C4—C5	1.377 (8)	C19—H19A	0.9600
C5—C6	1.372 (8)	C20—H20C	0.9600
C6—C7	1.381 (7)	C20—H20A	0.9600
C8—C10	1.493 (6)	C20—H20B	0.9600
C8—C9	1.494 (7)		
			
Cl1—Cu1—O1	108.38 (10)	C13—C12—C17	117.8 (4)
Cl1—Cu1—O3	108.44 (11)	C11—C12—C17	117.5 (4)
Cl1—Cu1—N2	96.87 (11)	C12—C13—C14	120.2 (3)
Cl1—Cu1—N4	89.26 (11)	O4—C13—C14	121.9 (4)
O1—Cu1—O3	143.06 (14)	O4—C13—C12	117.9 (4)
O1—Cu1—N2	81.01 (13)	C13—C14—C15	120.2 (4)
O1—Cu1—N4	96.61 (13)	C14—C15—C16	120.5 (4)
O3—Cu1—N2	97.49 (13)	C15—C16—C17	119.2 (4)
O3—Cu1—N4	80.97 (12)	C12—C17—C16	122.1 (4)
N2—Cu1—N4	173.85 (15)	C19—C18—C20	119.1 (4)
Cl1—Cu2—Cl2	99.52 (5)	N4—C18—C19	121.4 (4)
Cl1—Cu2—Cl3	135.68 (6)	N4—C18—C20	119.5 (4)
Cl1—Cu2—Cl4	94.92 (5)	H1WA—O1W—H1WB	104.00
Cl2—Cu2—Cl3	98.34 (5)	C3—C4—H4A	120.00
Cl2—Cu2—Cl4	137.89 (5)	C5—C4—H4A	120.00
Cl3—Cu2—Cl4	98.32 (5)	C6—C5—H5	119.00
Cu1—Cl1—Cu2	135.00 (5)	C4—C5—H5	119.00
Cu1—O1—C1	113.7 (3)	C5—C6—H6	120.00
Cu1—O3—C11	114.4 (2)	C7—C6—H6	120.00
N2—N1—C1	117.5 (3)	C6—C7—H7	119.00
Cu1—N2—N1	108.9 (3)	C2—C7—H7	119.00
Cu1—N2—C8	133.5 (3)	C8—C9—H9A	110.00
N1—N2—C8	117.6 (4)	C8—C9—H9C	109.00
C3—O2—H2	109.00	H9A—C9—H9B	109.00
N4—N3—C11	116.9 (3)	H9A—C9—H9C	109.00
Cu1—N4—N3	108.7 (3)	H9B—C9—H9C	109.00
N3—N4—C18	118.5 (4)	C8—C9—H9B	109.00
Cu1—N4—C18	132.6 (3)	C8—C10—H10A	109.00
C13—O4—H4	109.00	C8—C10—H10C	109.00
O1—C1—N1	118.9 (4)	H10A—C10—H10B	110.00
N2—N1—H1	121.00	C8—C10—H10B	109.00
C1—N1—H1	121.00	H10B—C10—H10C	109.00
N1—C1—C2	120.9 (3)	H10A—C10—H10C	109.00
O1—C1—C2	120.3 (4)	C13—C14—H14	120.00
C1—C2—C7	117.7 (3)	C15—C14—H14	120.00
C3—C2—C7	118.7 (4)	C16—C15—H15	120.00
C1—C2—C3	123.6 (4)	C14—C15—H15	120.00
O2—C3—C4	122.1 (4)	C15—C16—H16	120.00
C2—C3—C4	119.4 (4)	C17—C16—H16	120.00
C11—N3—H3	122.00	C16—C17—H17	119.00
O2—C3—C2	118.5 (4)	C12—C17—H17	119.00
N4—N3—H3	122.00	C18—C19—H19A	110.00
C3—C4—C5	120.1 (5)	C18—C19—H19B	109.00
C4—C5—C6	121.4 (5)	H19A—C19—H19B	110.00
C5—C6—C7	119.4 (5)	H19A—C19—H19C	110.00
C2—C7—C6	121.1 (4)	C18—C19—H19C	109.00
C9—C8—C10	118.3 (4)	H19B—C19—H19C	109.00
N2—C8—C9	122.7 (4)	C18—C20—H20B	110.00
N2—C8—C10	119.0 (4)	C18—C20—H20C	110.00
O3—C11—N3	118.9 (4)	C18—C20—H20A	110.00
N3—C11—C12	120.2 (4)	H20A—C20—H20C	109.00
O3—C11—C12	120.9 (3)	H20B—C20—H20C	109.00
C11—C12—C13	124.7 (3)	H20A—C20—H20B	109.00
			
O1—Cu1—Cl1—Cu2	119.83 (11)	C11—N3—N4—Cu1	2.0 (5)
O3—Cu1—Cl1—Cu2	−63.23 (12)	C11—N3—N4—C18	177.7 (4)
N2—Cu1—Cl1—Cu2	37.05 (13)	N4—N3—C11—O3	−3.7 (6)
N4—Cu1—Cl1—Cu2	−143.46 (12)	N4—N3—C11—C12	176.6 (4)
Cl1—Cu1—O1—C1	−95.8 (3)	Cu1—N4—C18—C19	176.3 (4)
O3—Cu1—O1—C1	89.0 (4)	Cu1—N4—C18—C20	−6.5 (7)
N2—Cu1—O1—C1	−1.5 (3)	N3—N4—C18—C19	1.8 (7)
N4—Cu1—O1—C1	172.8 (3)	N3—N4—C18—C20	179.0 (4)
Cl1—Cu1—O3—C11	−87.9 (3)	O1—C1—C2—C3	177.4 (4)
O1—Cu1—O3—C11	87.3 (3)	O1—C1—C2—C7	−4.3 (7)
N2—Cu1—O3—C11	172.3 (3)	N1—C1—C2—C3	−2.3 (7)
N4—Cu1—O3—C11	−1.7 (3)	N1—C1—C2—C7	176.0 (4)
Cl1—Cu1—N2—N1	110.0 (3)	C1—C2—C3—O2	−0.7 (7)
Cl1—Cu1—N2—C8	−68.4 (4)	C1—C2—C3—C4	179.5 (4)
O1—Cu1—N2—N1	2.4 (3)	C7—C2—C3—O2	−179.0 (4)
O1—Cu1—N2—C8	−176.0 (5)	C7—C2—C3—C4	1.2 (7)
O3—Cu1—N2—N1	−140.3 (3)	C1—C2—C7—C6	−179.4 (5)
O3—Cu1—N2—C8	41.3 (5)	C3—C2—C7—C6	−1.0 (7)
Cl1—Cu1—N4—N3	108.6 (3)	O2—C3—C4—C5	179.8 (5)
Cl1—Cu1—N4—C18	−66.3 (4)	C2—C3—C4—C5	−0.5 (8)
O1—Cu1—N4—N3	−143.0 (3)	C3—C4—C5—C6	−0.6 (9)
O1—Cu1—N4—C18	42.2 (5)	C4—C5—C6—C7	0.8 (9)
O3—Cu1—N4—N3	−0.2 (3)	C5—C6—C7—C2	0.0 (8)
O3—Cu1—N4—C18	−175.1 (5)	O3—C11—C12—C13	−177.9 (4)
Cl2—Cu2—Cl1—Cu1	−55.79 (9)	O3—C11—C12—C17	0.9 (7)
Cl3—Cu2—Cl1—Cu1	56.67 (11)	N3—C11—C12—C13	1.9 (7)
Cl4—Cu2—Cl1—Cu1	163.90 (8)	N3—C11—C12—C17	−179.3 (4)
Cu1—O1—C1—N1	0.2 (5)	C11—C12—C13—O4	1.0 (7)
Cu1—O1—C1—C2	−179.5 (3)	C11—C12—C13—C14	−179.6 (4)
Cu1—O3—C11—N3	3.4 (5)	C17—C12—C13—O4	−177.8 (4)
Cu1—O3—C11—C12	−176.8 (3)	C17—C12—C13—C14	1.6 (7)
C1—N1—N2—Cu1	−3.2 (5)	C11—C12—C17—C16	−179.3 (4)
C1—N1—N2—C8	175.5 (4)	C13—C12—C17—C16	−0.4 (7)
N2—N1—C1—O1	2.2 (6)	O4—C13—C14—C15	177.4 (4)
N2—N1—C1—C2	−178.2 (4)	C12—C13—C14—C15	−2.1 (7)
Cu1—N2—C8—C9	177.6 (4)	C13—C14—C15—C16	1.3 (7)
Cu1—N2—C8—C10	−2.2 (7)	C14—C15—C16—C17	−0.1 (7)
N1—N2—C8—C9	−0.7 (7)	C15—C16—C17—C12	−0.3 (7)
N1—N2—C8—C10	179.5 (4)		

### [Trichloridocopper(II)]-µ-chlorido-{bis[2-hydroxy-N'-(propan-2-ylidene)benzohydrazide]copper(II)} monohydrate Hydrogen-bond geometry (Å, º)

**Table:**

*D*—H···*A*	*D*—H	H···*A*	*D*···*A*	*D*—H···*A*
O2—H2···O1*W*	0.82	1.79	2.606 (6)	171
N1—H1···O2	0.86	1.94	2.597 (5)	132
N3—H3···O4	0.86	1.96	2.612 (5)	132
O1*W*—H1*WA*···O3^i^	0.85	2.53	3.375 (6)	170
O1*W*—H1*WA*···N3^i^	0.85	2.59	3.303 (7)	142
O1*W*—H1*WB*···Cl3^ii^	0.85	2.38	3.195 (6)	160
O4—H4···Cl2^iii^	0.82	2.40	3.215 (3)	175
C7—H7···O1	0.93	2.44	2.762 (5)	1
C10—H10*C*···O3	0.96	2.35	3.099 (6)	135
C17—H17···O3	0.93	2.43	2.760 (5)	101
C20—H20*C*···O1	0.96	2.41	3.043 (5)	123

Symmetry codes: (i) −*x*+2, −*y*+1, −*z*+1; (ii) −*x*+1, −*y*+1, −*z*+1; (iii) *x*+1/2, −*y*+3/2, *z*−1/2.
